# Value of *Plasmodium falciparum* Histidine-Rich Protein 2 Level and Malaria Retinopathy in Distinguishing Cerebral Malaria From Other Acute Encephalopathies in Kenyan Children

**DOI:** 10.1093/infdis/jit500

**Published:** 2013-09-16

**Authors:** Symon M. Kariuki, Evelyn Gitau, Samson Gwer, Henry K. Karanja, Eddie Chengo, Michael Kazungu, Britta C. Urban, Charles R. J. C. Newton

**Affiliations:** 1 Center for Geographic Medicine Research Coast, Kenya Research Institute, Kilifi; 2 Department of Medical Physiology, College of Health Sciences, Kenyatta University; 3 Clinical Research, Afya Research Africa, Nairobi, Kenya; 4 Department of Psychiatry, University of Oxford; 5 Liverpool School of Tropical Medicine; 6 Neurosciences Unit, Institute of Child Health, University College London, United Kingdom

**Keywords:** attributable fractions, cerebral malaria, children, histidine-rich protein-2, malaria retinopathy

## Abstract

***Background.*** The diagnosis of cerebral malaria is problematic in malaria-endemic areas because encephalopathy in patients with parasitemia may have another cause. Abnormal retinal findings are thought to increase the specificity of the diagnosis, and the level of histidine-rich protein 2 (HRP2) may reflect the parasite biomass.

***Methods.*** We examined the retina and measured plasma HRP2 levels in children with acute nontraumatic encephalopathy in Kenya. Logistic regression, with HRP2 level as an independent variable and World Health Organization–defined cerebral malaria and/or retinopathy as the outcome, was used to calculate malaria-attributable fractions (MAFs) and retinopathy-attributable fractions (RAFs).

***Results.*** Of 270 children, 140 (52%) had peripheral parasitemia, 80 (30%) had malaria retinopathy, and 164 (61%) had an HRP2 level of >0 U/mL. During 2006–2011, the incidence of HRP2 positivity among admitted children declined by 49 cases per 100 000 per year (a 78% reduction). An HRP2 level of >0 U/mL had a MAF of 93% for cerebral malaria, with a MAF of 97% observed for HRP2 levels of ≥10 U/mL (the level of the best combined sensitivity and specificity). HRP2 levels of >0 U/mL had a RAF of 77% for features of retinopathy combined, with the highest RAFs for macular whitening (99%), peripheral whitening (98%), and hemorrhages (90%).

***Conclusion.*** HRP2 has a high attributable fraction for features of malarial retinopathy, supporting its use in the diagnosis of cerebral malaria. HRP2 thresholds improve the specificity of the definition.

Cerebral malaria is the most severe neurological complication of falciparum malaria and is associated with significant morbidity and mortality [1]. The World Health Organization (WHO) defines cerebral malaria as unarousable coma (Blantyre coma score of ≤2 or inability to localize a painful stimulus) in a child with asexual malaria parasites in the peripheral blood and exclusion of other causes of encephalopathy [2]. In most malaria-endemic areas, this definition is problematic for 2 reasons. First, the prevalence of parasitemia in asymptomatic children in the community can be as high as 70%, and thus a child admitted with coma from any cause may have coincidental parasitemia [3]. This proportion of children with asymptomatic parasitemia increases with an increase in transmission intensity of malaria and vice versa [4]. Second, the exclusion of potential causes of childhood nontraumatic coma requires an extensive battery of tests, including virologic and neuroimaging analyses, most of which are not available in hospitals in malaria-endemic areas. These problems with the definition of malaria can affect interventions such as vaccines and treatment studies [5].

The pathognomonic feature of pathological cerebral malaria is sequestration of parasite-infected erythrocytes in the cerebral microvasculature. Taylor et al found that 23% of Malawian children who died after fulfilling WHO-defined criteria for cerebral malaria antemortem did not have sequestered parasite-infected erythrocytes [6]. They found that malaria retinopathy (ie, retinal whitening [macular and peripheral], vessel color changes, and retinal hemorrhages with or without papilledema) was strongly associated with parasite-infected erythrocytes sequestered in the brain [7]. The retinal findings may reflect those in the brain since the retina and the cerebral cortex are embryologically, anatomically, and physiologically related [8]. Although such findings are based on small numbers of children who died, they demonstrated that malaria retinopathy can be used in evaluating the diagnostic usefulness of other clinical laboratory features in sequestration-defined cerebral malaria. There is a need to identify other clinical and laboratory measures to distinguish cerebral malaria from other encephalopathies because autopsy studies reflect the situation in the fatal cases only and may not be representative of children who survive and because peripheral parasitemia may not reflect the parasites sequestered in the brain [1].

Histidine-rich protein 2 (HRP2) is secreted by *Plasmodium falciparum* throughout its 48-hour cycle and remains intraerythrocytic until mature schizonts release it into blood [9]. It is thought to reflect the parasitemia biomass and to have diagnostic and prognostic value in malaria [10, 11]. However, the diagnostic value of HRP2 in cerebral malaria has not been validated. Recently, Hendriksen et al used a mechanistic model to show that HRP2 reliably identified those with increased risk for coma [12, 13], but they did not evaluate the diagnostic value of HRP2 levels in patients with retinopathy. Only 1 study attempted to validate HRP2 against malarial retinopathy by using receiver operating characteristic (ROC) curve statistics, and it found that HRP2 accounted for 90% of the area under the ROC curve [14]. However, ROC curve statistics are less sensitive than likelihood tests and are less likely to distinguish the relative contribution of many risk factors affecting an outcome [15].

Logistic regression techniques that use maximum likelihood analysis [16] can be used to compute the malaria-attributable fraction (MAF) for WHO-defined cerebral malaria and the retinal attributable fraction (RAF) for cerebral malaria, using the HRP2 level as a marker of sequestered biomass. Attributable fractions are considered predictive estimates of the number of children who would not be admitted with cerebral malaria if malaria were eliminated. Logistic regression techniques compute attributable fractions by comparing HRP2 levels in children admitted with WHO-defined cerebral malaria and/or retinopathy (the case group) to that of children admitted without these features (the comparison group) [17]. Logistic regression uses the distribution of HRP2 levels to compute attributable fractions that are both sensitive and specific, as opposed to the dichotomous presence or absence of HRP2, which would be biased toward sensitivity [17]. We recently used a similar method to define and estimate the proportion of seizures attributable to falciparum malaria in a malaria-endemic area on the Kenyan coast [18].

We used a logistic model to determine the diagnostic value of HRP2 levels by computing attributable fractions for WHO-defined cerebral malaria and features of retinopathy. We further examined whether the incidence of HRP2 positivity among admitted children declined during a period when the incidence of malaria decreased on the Kenyan coast.

## METHODS

### Patients and Study Area

This study was conducted in Kilifi District Hospital (KDH) in a malaria-endemic area on the Kenyan coast. KDH is the main district-level hospital in the area and draws its admissions predominantly from the local residents, who are from the Mijikenda community. The hospital morbidity surveillance is directly linked with a population framework referred to as the Kilifi Health Demographic Surveillance System (KHDSS) [19]. This area has seen a significant reduction in hospital admissions with malaria and parasite prevalence in the community [20], both of which are good surrogate measures for a changing malaria transmission intensity. The hospital has a 35-bed pediatric ward and an 8-patient high-dependency unit that mainly admit children with malaria and bacterial infections [19]. The present analysis is part of larger ongoing study that is investigating the pathophysiology of severe malaria under the auspice of the Severe Malaria in African Children network [21]. Study participants were all children admitted with coma (defined as a Blantyre coma score of ≤2 or the inability to localize a painful stimuli) [22] between July 2005 and December 2011. These children were given appropriate emergency care and treatments according to the national guidelines by clinicians and nurses at the hospital, who provide 24-hour clinical cover.

### Clinical and Laboratory Procedures

We prospectively collected standardized clinical, laboratory, and anthropometric data for this study. Clinicians were trained in an indirect ophthalmoscopy by an experienced ophthalmologist. They performed ophthalmoscopy on all children older than 9 months who presented with encephalopathy, defined as inability to localize a painful stimulus [23]. The signs of malarial retinopathy can be identified in unconscious children by trained clinicians using indirect fundoscopy, which has a sensitivity of 95% [24]. Pupils were dilated with tropicamide 0.5% or 1% eye drops, with the addition of 2.5% phenylephrine if required. The 2 clinicians (E. C. and M. K.) who conducted most (80%) of the examinations had a substantial agreement for the features of retinopathy (κ = 0.70). Complete blood counts were performed using an automated system (Beckman/Counter), and blood gas concentration was determined by the IL 1620 analyzer (Instrument Laboratories). Falciparum malaria parasitemia was counted on thick and thin blood slides stained with 10% Giemsa.

### Determination of HRP2 Levels

An enzyme-linked immunosorbent assay (ELISA) was used to determine the presence of HRP2 in frozen plasma samples, which had been stored at −80°C for 1–7 years. High-adsorbing plates (Maxisorp NUNC-Immuno plate) were coated with 100 µL/well of 1.0 µg/mL immunoglobulin M monoclonal anti-HRP2 antibody (MPFM-55A, Immunology Consultants Laboratories, Newberg, OR), diluted in phosphate-buffered saline (PBS), and incubated overnight at 4°C. One hundred microliters of diluted plasma samples (1:64) were added to the plates, and plates were sealed and incubated at room temperature in a humid chamber for 2 hours and then washed 5 times. One hundred microliters of secondary antibody conjugated with horseradish peroxidase (MPFG-55P, Immunology Consultants Laboratories; 0.2 μg/mL diluted in 2% bovine serum albumin, 1% Tween 20, and PBS) was added to the wells and incubated in a humid chamber for another 1 hour at room temperature. Substrate (SigmaFast OPD P9187-50SET) was then added, incubation was performed for 30 minutes at room temperature, and the reaction was stopped by adding 50 µL of 2 N sulfuric acid to each well. The plates were read using a plate reader at an OD of 490. The standards were made by serially diluting plasma samples of known parasitemia, with the highest parasitemia at 0.2% and the lowest at 0.003125% (this gave a 0.1 absorbance value above the negative absorbance). HRP2 levels are specified in arbitrary units per milliliter (U/mL) of plasma, because no recombinant protein was used.

### Definition of Terms

Cerebral malaria was defined as unarousable coma in a child admitted with slide-positive falciparum malaria and whose coma did not resolve 30 minutes after cessation of seizures or correction of electrolyte imbalances or hypoglycemia [25]. The retinopathy definition included hemorrhages, peripheral whitening, macular whitening, papilledema, or vessel color changes [24]. Respiratory distress was defined as deep Kussmaul-type breathing. Epilepsy was defined as history of 2 unprovoked seizures, meningitis was defined as a cerebrospinal fluid white blood cell count of >50 cells/µL, sepsis was defined as evidence of inflammation and microbial process, and undetermined encephalopathy was defined as coma whose cause could not be established. Malnutrition was defined as a height-for-age *z* score of ≤2, severe anemia was defined as a hemoglobin concentration of <50 g/L, hypoglycemia was defined as a blood glucose concentration of <3 mmol/L, and hyponatremia was defined as a sodium concentration of <135 mEq/L. Acidosis was defined as a base excess value of ≤8. Parasitemia was determined by standard methods [26]. On admission, clinicians enquired about premorbid conditions.

### Statistical Analysis

All analyses were performed using Stata (version 11, Stata Corp, College Station, TX). The Mann-Whitney *U* test was used to compare age between retinopathy-positive admissions and retinopathy-negative admissions. Pearson χ^2^ test was used to compare categorical variables between groups. The *z* scores for malnutrition were computed using a Stata command *zanthro* (2000 CDC Growth Reference, USA). Only the ﬁrst hospital admission with HRP-positive coma was considered in the incidence analysis. The incidence was calculated for children who lived in the KHDSS, using the estimated midyear population count of children as the denominator. A decline in the incidence of these admissions was estimated as the difference between mean incidence for the first 2 years of the study and the mean incidence for the last 2 years of the study.

Logistic regression methods were used to model the risk of WHO-defined cerebral malaria and/or retinopathy as a continuous function of HRP2 level in these children [17]. Absolute HRP2 levels in children with WHO-defined cerebral malaria and/or retinopathy and from a comparison group of children without these conditions were used to model the relationship between HRP2 levels and cerebral malaria and/or retinopathy.

We used the logistic regression model log[*p*/1–*p*] =[ *a* + *bx^t^*], where *p* is the probability that a child with HRP2 at density *x* has cerebral malaria/malarial retinopathy, and *t* is the power function that stabilizes the maximum likelihood estimation. The coefficient *b* was then used to calculate the MAF for WHO-defined cerebral malaria and the RAF for sequestration-defined cerebral malaria for each child, first at any HRP2 level and then at different HRP2 thresholds. The risks of a group with cerebral malaria and/or retinopathy were averaged to estimate the proportion of the group whose encephalopathy is caused by malaria rather than by another illness with coincidental HRP2. The averaged risks gave the MAF for WHO-defined cerebral malaria or the RAF for sequestration-defined cerebral malaria for the group analyzed [16], as defined by HRP2. Sensitivity and speciﬁcity were then calculated using the MAF for retinopathy or the proportion of cases with retinopathy within each level of HRP2. This logistic regression analysis was repeated for specific features of retinopathy. Confidence intervals (CIs) around the MAF were estimated by bootstrapping with 500 iterations. The logistic model was adjusted for age (categorized into those older and those younger than 5 years, a cutoff age when immunity to severe malaria develops [27]) and year (as a surrogate measure of changing transmission intensity of malaria). A *P* value of ≤.05 was considered statistically significant.

Permission to conduct this study was granted by the Kenyan Medical Research Institute National Ethics Committee (Scientific Steering Committee no. 1249).

## RESULTS

**Figure 1.**
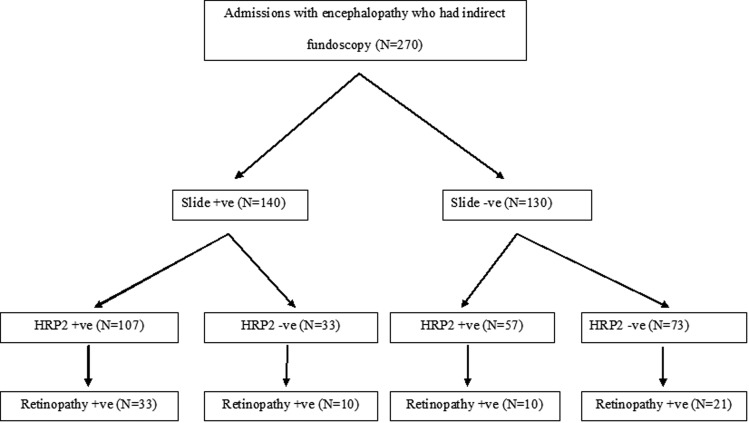
Flowchart of slide-positive, histidine-rich protein 2 (HRP2) positivity and retinopathy positivity among children admitted with encephalopathy. Parasitemia and malaria retinopathy were examined on the day of admission, whereas assays of HRP2 were done in the last year of the study.

Indirect fundoscopy was performed in 270 children admitted with acute encephalopathy to KDH. Of the children who met the WHO definition for cerebral malaria, 164 (60.7%) were positive for HRP2 in plasma, and 140 (51.9%) had peripheral parasitemia (Figure 1). At least 1 feature of retinopathy was observed in 80 of 270 study participants (29.6%). Of the 270 study participants, 147 (54.4%) were males. The median age was 39.0 months (interquartile range, 25.0–55.0 months) and was significantly higher among slide-positive admitted children than slide-negative admitted children, but there was no difference between retinopathy-positive and retinopathy-negative admitted children (Table 1).

**Table 1.** Clinical and Laboratory Characteristics of Admissions With Acute Nontraumatic Encephalopathy

Characteristic	Parasitemia Status				Retinopathy Status			
	Positive	Negative	Statistic or Score	*P*	Positive	Negative	Statistic or Score	*P*
Age, mo	41 (29–53)	35 (16–57)	*z* = −2.21	.0275	40 (29–56)	39 (21–54)	*z* = −1.06	.2898
Male sex	72/138 (52.2)	75/127 (59.1)	χ^2^ = 1.27	.260	46/79 (58.2)	101/186 (54.3)	χ^2^ = 0.35	.5560
Delivered at hospital	8/124 (6.5)	13/110 (11.8)	χ^2^ = 2.06	.1520	9/71 (12.7)	12/163 (7.4)	χ^2^ = 1.71	.1910
Head circumference	48.6 (47.0–50.0)	48.0 (45.1–49.7)	*z* = −1.73	.0831	48.0 (46.3–49.5)	48.0 (46.0–50.0)	χ^2^ = 0.104	.9175
Jaundice	6/125 (4.8)	6/120 (5.0)	χ^2^ = 0.01	.9420	6/75 (8.0)	6/170 (3.5)	χ^2^ = 2.23	.1350
Perinatal hospitalization	3/99 (3.0)	6/92 (6.5)	χ^2^ = 1.29	.255	0/63 (0.0)	9/128 (7.0)	χ^2^ = 4.65	.0310
Prematurity	3/99 (3.0)	2/93 (2.2)	χ^2^ = 0.15	.702	5/124 (4.0)	0/64 (1.2)	χ^2^ = 2.65	.1030
Birth problems	16/140 (11.4)	11/130 (8.5)	χ^2^ = 0.66	.417	9/80 (11.3)	18/190 (9.5)	χ^2^ = 0.197	.6570
Malnutrition	42/120 (35.0)	64/105 (61.0)	χ^2^ = 15.14	<.0001	30/70 (43.9)	76/155 (49.0)	χ^2^ = 0.74	.3390
Respiratory distress	26/137 (19.0)	35/127 (27.6)	χ^2^ = 2.73	.098	14/79 (17.7)	47/185 (25.4)	χ^2^ = 1.84	.1750
Febrile temperatures	113/124 (91.3)	100/120 (83.3)	χ^2^ = 3.34	.068	70/75 (93.3)	143/169 (84.6)	χ^2^ = 3.56	.0590
Seizures	110/123 (89.4)	92/100 (92.0)	χ^2^ = 0.43	.5140	59/65 (90.8)	143/158 (90.5)	χ^2^ = 0.01	.9510
Severe anemia	14/111 (12.6)	4/80 (5.0)	χ^2^ = 3.16	.076	8/53 (15.1)	10/138 (7.3)	χ^2^ = 2.8	.0960
Acidosis	55/102 (53.9)	29/65 (44.6)	χ^2^ = 1.38	.241	24/44 (54.6)	60/123 (48.8)	χ^2^ = 0.43	.5120
Leukocytosis	52/140 (37.1)	45/129 (34.9)	χ^2^ = 0.15	.700	31 (38.8)	66/189 (34.9)	χ^2^ = 0.36	.5550
Thrombocytopenia	76/111 (68.5)	6/80 (7.5)	χ^2^ = 70.54	<.0001	34/56 (60.7)	48/135 (35.6)	χ^2^ = 10.23	.0010
Hypoglycemia	14/140 (10.0)	9/130 (6.9)	χ^2^ = 0.82	.3650	10/80 (12.5)	13/190 (6.8)	χ^2^ = 2.31	.1280
Hyponatremia	46/97 (47.4)	36/77 (46.8)	χ^2^ = 0.01	.930	22/52 (42.3)	60/122 (49.2)	χ^2^ = 0.69	0.401
HRP2 level	19 764 (2872–28 802)	27 (0–283)	*z* = −7.25	<.0001	26 969 (3223–31 523)	578 (4.5–18 799)	*z* = −4.74	<.0001
Geometric mean parasitemia (95% CI)	32 095 (1898–54 259)	…	…		2031 (8761–47 053)	27 830 (14 928–51 883)	*t* = 0.42	.6764
Died	21/140 (15.0)	39/130 (30.0)	χ^2^ = 8.78	.003	21/80 (26.3)	39/190 (20.5)	χ^2^ = 1.07	.3020

Data are proportion (%) of patients or median level (interquartile range), unless otherwise indicated. See Methods for definitions.

Abbreviations: CI, confidence interval; Hb, hemoglobin; HRP2, histidine-rich protein 2; WBC, white blood cell.

### Clinical and Laboratory Characteristics Associated With Encephalopathy

**Figure 2.**
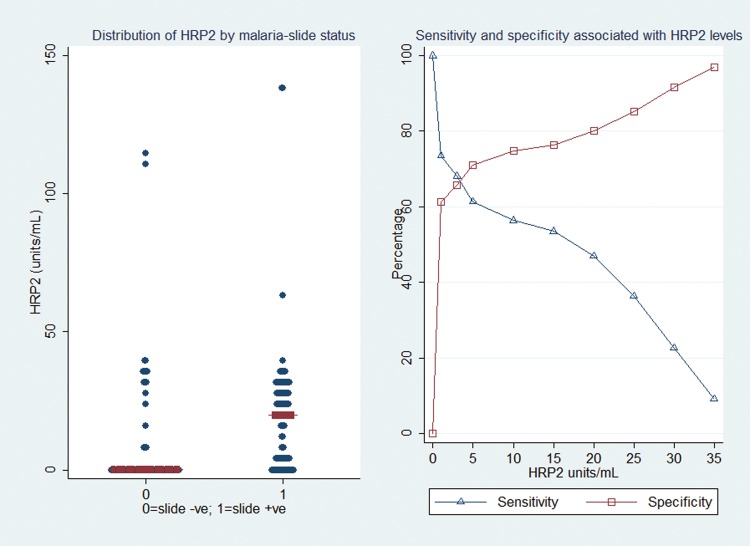
The median and distribution of histidine-rich protein 2 (HRP2) levels among admitted children with acute encephalopathy, by malaria and/or retinopathy status, and the sensitivities and specificities associated with HRP2 levels among admitted children with World Health Organization–defined cerebral malaria. The median levels of HRP2 were significantly higher among children with than among those without a slide positive for malaria parasites, and levels were higher among children with than among those without retinopathy. Sensitivities worsened with higher levels of HRP2, whereas specificities improved.

Results of comparisons between children who were positive and those who were negative for parasitemia and/or retinopathy are shown in Table 1. The median HRP2 level was significantly greater among children with than among those without slide-positive encephalopathy (*P* < .0001) and significantly greater among children with than among those without retinopathy (*P* < .0001; Figure 2 and Table 1). Thrombocytopenia was more common among children with than among those without slide-positive encephalopathy (*P* < .0001) and more common among children with than among those without retinopathy (*P* = .001). Malnutrition was less common among slide-positive admitted children than among slide-negative admitted children (*P* < .0001). Additionally, previous hospitalization during the perinatal period was less common among children with than among those without retinopathy (*P* = .028). The median levels of HRP2 were higher among children with retinopathy than among those without retinopathy for all features except vessel color changes (Table 2).

**Table 2.** Retinopathy Status of Study Participants and Associated Median Histidine-Rich Protein 2 Levels

Feature	Retinopathy- Positive Participants		Retinopathy- Negative Participants		*P*^a^
	No.	HRP2 Level, Median (IQR)	No.	HRP2 Level, Median (IQR)	
At least 1 sign of retinopathy	80	27 (3–32)	190	0.6 (0.005–19)	<.0001
Hemorrhages	41	29 (22–34)	190	0.6 (0.005–19)	<.0001
Macular whitening	21	31 (28–34)	190	0.6 (0.005–19)	<.0001
Papilledema	24	31 (1–36)	190	0.6 (0.005–19)	.0129
Peripheral whitening	15	30 (23–32)	190	0.6 (0.005–19)	.0001
Vessel color changes	18	14 (0–30)	190	0.6 (0.005–19)	.3640

Abbreviation: IQR, interquartile range.

^a^ For the median difference in HRP2 level between the retinopathy-positive and retinopathy-negative groups. The retinopathy features are not mutually exclusive. HRP2 levels are arbitrary units per microlitre of plasma.

### Retinopathy Among Admitted Children With Encephalopathy

Of the 270 children who had indirect fundoscopic examination performed, 80 (29.6%) had at least 1 feature of retinopathy: hemorrhages were observed in 41 (51·3%), papilledema in 24 (30.0%), macular whitening in 21 (26.3%), vessel color changes in 18 (22.5%), and peripheral whitening in 15 (18.8%; Table 2).

### MAFs and RAFs for Admitted Children With Encephalopathy

**Figure 3.**
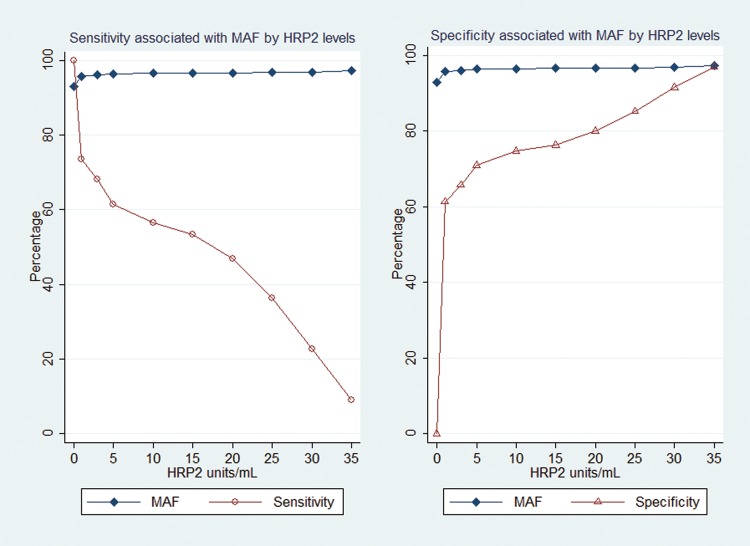
Sensitivity and specificity of malaria-attributable fractions for children admitted with encephalopathy, by histidine-rich protein 2 (HRP2) level. MAFs were first determined at HRP2 levels of >0 U/mL and then at HRP2 levels with the best combined sensitivity and specificity.

HRP2 levels of >0 U/mL had a MAF of 92.9% (95% CI, 83.9%–97.8%) for WHO-defined cerebral malaria; the MAF was 96.0% (95% CI, 89.5–99.2) for ≥3 U/mL, the threshold with the best combined sensitivity and specificity (Figures 2 and 3). Use of higher HRP2 thresholds further reduced the sensitivity of the case definition.

HRP2 levels of >0 U/mL had a RAF of 76.7% (95% CI, 52.1%–90.9%) for at least 1 feature of retinopathy; the RAF increased to 83.6% (95% CI, 59.3%–95.1%) for levels of ≥5 U/mL, the threshold for the maximum combined sensitivity and specificity. Higher levels of HRP2 reduced the sensitivity.

For macular whitening, HRP2 levels of >0 U/mL had a RAF of 98.8% (95% CI, 87.2%–100.0%); levels of ≥5 U/mL had a RAF of 99.8% (95% CI, 92.7%–100.0%) and the best combined sensitivity and specificity (Table 3). For peripheral whitening, HRP2 levels of >0 U/mL had a RAF of 97.7% (95% CI, 71.1%–100.0%); levels of ≥5 U/mL had a RAF of 99.4% (95% CI, 79.6%–100.0%) and the best combined sensitivity and specificity. For hemorrhages, HRP2 levels of >0 U/mL had a RAF of 90.1% (95% CI, 67.2%–98.5%); levels of ≥5 U/mL had a RAF of 94.6% (95% CI, 74.5%–99.6%) and the best combined sensitivity and specificity. HRP2 levels had a low RAF for papilledema and vessel color changes (Table 3).

**Table 3.** Malaria-Attributable Fractions and Retinopathy Attributable Fractions, by Histidine-Rich Protein 2 (HRP2) Level, Among Children Admitted With Nontraumatic Acute Encephalopathy

Category	Attributable Fraction, % (95% Confidence Interval), by HRP2 Level									
	>0 U/mL		≥5 U/mL		≥10 U/mL		≥20 U/mL		≥30 U/mL	
WHO-defined cerebral malaria	92.9 (83.9–97.8)		96.4 (90.0–99.3)		96.5 (90.3–99.4)		96.7 (90.5–99.4)		96.9 (90.9–99.5)	
At least one retinopathy feature	76.7%	52.1%–90.9%	83.6%	59.3%–95.1%	84.0%	59.6%–95.3%	84.3%	60.0%–95.5%	85.1%	60.9%–95.8%
Peripheral whitening	97.7%	71.1%–100.0%	99.4%	79.6%–100.0%	99.5%	80.0%–100.0%	99.5%	80.4%–100.0%	99.6%	81.0%–100.0%
Macular whitening	98.8%	87.2%–100.0%	99.8%	92.7%–100.0%	99.8%	93.4%–100·0%	99.8%	93.5%–100·0%	99.8%	93.6%–100·0%
Hemorrhages	90.1%	67.2%–98.5%	94.6%	74.5%–99.7%	94.8%	74.7%–99.7%	94.9%	75.0%–99.7%	95.2%	74.8%–99.8%
Papilledema	67.2%	0%–95.6%	75.0%	0%–98.9%	75.5%	0%–99.0%	76.0%	0%–99.1%	76.8%	0%–99.2%
Vessel color changes^a^	0%	0%–79.9%	0%	0%–87.4%	0%	0%–87.7%	0%	0%–88.0%	0%	0%–88.7%

All AFs were adjusted for age and year of study.

Abbreviations: CI, confidence interval; WHO, World Health Organization.

^a^ All AFs and the lower levels of their 95% CIs were ≤0, but the upper levels of their 95% CIs were large. The lower levels of the 95% C1s of the AF for papilledema were ≤0.

### Frequency of Other Potential Causes of Coma, by HRP2 Thresholds

The following alternative causes of coma were more common among children with HRP2 levels below the modeled thresholds (ie, 10 U/mL), compared with children with greater HRP2 levels, for WHO-defined cerebral malaria: meningitis, 19 of 22 (86.3%) versus 2 of 22 (13.7%); undetermined encephalopathy, 37 of 49 (75.5%) versus 12 of 39 (24.5%); epilepsy, 3 of 5 (60.0%) versus 2 of 5 (40.0%); and sepsis, 2 of 3 (66.7%) versus 1 of 3 (33.3%).

### Decline in the Incidence of HRP2-Positive Acute Encephalopathy

**Figure 4.**
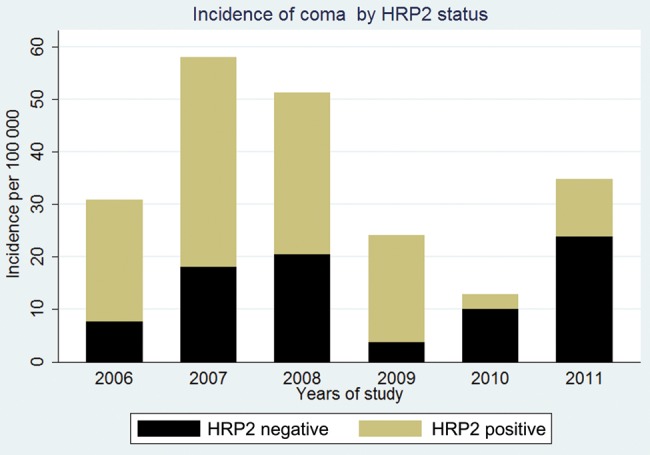
The declining incidence of histidine-rich protein 2 (HRP2) positivity among children admitted with coma during a period when the malaria incidence had declined. The incidence was calculated for children who lived in the Kilifi Health Demographic Surveillance System, using the annual midyear populations of children as the denominators.

The incidence of HRP2-positive encephalopathy among admitted children decreased by 78%, from 63 cases per 100 000 per year in 2006 and 2007 to 14 cases per 100 000 per year in 2010 and 2011 (Figure 4). This was slightly smaller than the modeled MAF of 93% for WHO-defined cerebral malaria at HRP2 levels of >0 U/mL.

## DISCUSSION

This study demonstrates that plasma HRP2 levels in children with encephalopathy improve the specificity of the diagnosis of cerebral malaria, as further supported by very high RAFs for hemorrhages and macular and peripheral whitening, the features of retinopathy-defined cerebral malaria. The usefulness of the HRP2 level in the diagnoses of malaria would be further improved by using a plasma level that offers a balance between sensitivity and specificity and the exclusion of alternative causes of coma. These conclusions are supported by the proportional decline in the incidence of HRP2 positivity among admitted children over the study period comparable to modeled MAFs and RAFs.

The median HRP2 levels among admitted children with coma were significantly higher for those who were positive for parasitemia and retinopathy than for those who were negative, supporting the hypotheses that parasite biomass is related to cerebral malaria. Additionally, children who were negative for retinopathy were more likely to have been previously hospitalized with perinatal complications, compared with children who were positive, suggesting that retinopathy is more related to malaria than other causes, as documented in Malawi [28]. Thrombocytopenia was more common among children with than among children without cerebral malaria and/or retinopathy, which is consistent with previous findings for severe malaria [29].

The high MAFs for WHO-defined cerebral malaria are comparable to those reported by another study [12], supporting the diagnostic role of HRP2. The computed MAF for WHO-defined cerebral malaria was comparable to the observed decline of HRP2 positivity among admitted children with encephalopathy over a period when the burden of malaria was reduced, although the observed decline was slightly smaller. Attributable fractions can be influenced by factors related to acquisition of immunity, such as age and transmission intensity of malaria [16]. In particular, low transmission intensity is associated with high MAF or RAF [17], but we attempted to account for this effect by including year of study and age into the logistic model. The smaller observed decline in the incidence of HRP2 positivity among admitted children may be ascribed to the increasing ratio of cerebral malaria to severe malaria anemia as the transmission intensity for malaria decreases [20].

The high RAF as explained by HRP2 level, albeit for all features of retinopathy (including those clinically known to be less specific to malaria) [24], further highlights the diagnostic value of the HRP2 level in cerebral malaria. Features of retinopathy specific to malaria may reflect a subgroup of cerebral malaria involving sequestration, as was demonstrated in an autopsy study [6].

HRP2 levels of >0 U/mL were associated with the highest RAF for retinal whitening (99% for macular whitening, 98% for peripheral whitening, and 90% for hemorrhages 90%). The RAF increased to almost 100% for macular and peripheral whitening and to 95% for the hemorrhages at an HRP2 level of 5 U/mL, which is a balance between both sensitivity and specificity. Retinal whitening may be more specific to malaria, since it is rarely seen in other childhood illnesses [30]. It is thought to be a result of oncotic swelling of neurons in the inner retina due to metabolic or hypoxic stress that occurs in cerebral malaria [24, 25].

At any level of HRP2, the RAF for hemorrhages was high (90%) but was expected to be lower than that for retinal whitening, since hemorrhages may be caused by other conditions, such as severe anemia [31]. The low RAF for vessel color changes at any HRP2 level is surprisingly low, since this feature would be expected to be more specific than hemorrhages to malaria [24]. Vessel color changes may have been missed on examination, which could have reduced the RAF [32]. In particular, incorrect judgment of retinal signs by the examiner (eg, choroidal vessels being mistaken for the abnormal vessels of cerebral malaria) may also explain the low RAF for vessel color changes. In Ghanaian and Malian studies [30, 33], vessel color changes were not common among children with cerebral malaria, suggesting regional differences in this sign [24].

These HRP2-determined attributable fractions computed in this study have implication in design of intervention trials for cerebral malaria. For example, the AQUAMAT study found that artesunate reduced mortality by 22.5%, compared with quinine [34], and this was based on correct use of a dichotomous presence or absence of lactate dehydrogenase, which may be as specific as HRP2. If the definition of cerebral malaria in this study relied on the presence of parasitemia only (which has a MAF of 85% [17], compared with 93% for HRP2 in this study), the relative mortality would have been underestimated as 20.5%.

The proportion of healthy community children with malarial retinopathy is not known, since it is technically difficult to perform indirect fundoscopy on conscious children, so we assumed that retinopathy in our study subjects was related to the hospital admission. The attributable fractions modeled are based on HRP2 data assessed with an ELISA that may not be 100% sensitive, and the long duration of storage of some samples may have affected the validity. Furthermore, the levels of HRP2 may have been affected for children who were treated with antimalarials in periphery facilities, which can further complicate modeling of attributable fractions.

This study confirms that the HRP2 level can reliably be used to distinguish cerebral malaria from other acute encephalopathies, since it was associated with high attributable fractions for cerebral malaria and/or retinopathy (particularly, retinal whitening and hemorrhages). Further inclusion of HRP2 thresholds and exclusion of alternative causes of coma would improve the specificity of the definition of cerebral malaria in intervention studies. The study also confirms that papilledema is not specific to malaria and that assessment of vessel color changes may require an experienced clinician, but there may be regional differences in this sign. These findings need to be replicated in other areas before the HRP2 level can be universally accepted for the diagnosis of cerebral malaria.
